# Computational comparison of anterior lumbar interbody fusion and oblique lumbar interbody fusion with various supplementary fixation systems: a finite element analysis

**DOI:** 10.1186/s13018-022-03480-z

**Published:** 2023-01-02

**Authors:** Pengrong Ouyang, Qinghua Tan, Xijing He, Bo Zhao

**Affiliations:** Department of Orthopedics, Second Affiliated Hospital of Xi’an Jiaotong University, Xi’an, Shaanxi Province China

**Keywords:** Anterior lumbar interbody fusion, Oblique lumbar interbody fusion, Spinal internal fixation device, Biomechanical properties, Finite element analysis

## Abstract

**Background and objective:**

Anterior lumbar interbody fusion (ALIF) and oblique lumbar interbody fusion (OLIF) have shown a great surgical potential, while it has always been controversial which surgical approach and which type of fixation system should be selected. This study investigated the biomechanical response of ALIF and OLIF with various supplementary fixation systems using the finite element method.

**Materials and methods:**

Lumbar L4–L5 ALIF and OLIF models stabilized by different supplementary fixation systems (stand-alone cage, integrated stand-alone cage, anterior plate, and bilateral pedicle screw) were developed to assess the segmental range of motion (ROM), endplate stress (EPS), and screw-bone interface stress (SBIS).

**Experimental results:**

ALIF showed lower ROM and EPS than OLIF in all motion planes and less SBIS in the most of motion planes compared with OLIF when the anterior plate or pedicle screw was used. ALIF induced higher ROM, while lower EPS and SBIS than OLIF in the majority of motion planes when integrated stand-alone cage was utilized. Using a stand-alone cage in ALIF and OLIF led to cage migration. Integrated stand-alone cage prevented the cage migration, whereas caused significantly larger ROM, EPS, and SBIS than other fixation systems except for the rotation plane. In the most of motion planes, the pedicle screw had the lowest ROM, EPS, and SBIS. The anterior plate induced a slightly larger ROM, EPS, and SBIS than the pedicle screw, while the differences were not significant.

**Conclusion:**

ALIF exhibited a better performance in postoperative segmental stability, endplate stress, and screw-bone interface stress than OLIF when the anterior plate or the pedicle screw was used. The pedicle screw could provide the greatest postoperative segmental stability, less cage subsidence incidence, and lower risk of fixation system loosening in ALIF and OLIF. The anterior plate could also contribute to the stability required and fewer complications, while not as effectively as the pedicle screw. Extreme caution should be regarded when the stand-alone cage is used due to the risk of cage migration. The integrated stand-alone cage may be an alternative method; however, further optimization is needed to reduce complications and improve postoperative segmental stability.

## Background

Posterior lumbar interbody fusion surgeries, such as posterior lumbar interbody fusion (PLIF) and transforaminal lumbar interbody fusion (TLIF), are the most commonly used lumbar interbody fusion surgical approaches in treating lumbar degenerative diseases. These surgical approaches possess the advantages of safety, familiarity to surgeons, and direct neural decompression [1]. However, they are accompanied by the morbidity of substantial bleeding, incidental durotomy, epidural adhesion, and paraspinal musculature injury [1]. Thus, orthopaedic surgeons have always been exploring an alternative minimally invasive surgical approach for lumbar interbody fusion.

The anterior and anterolateral approaches for lumbar interbody fusion, such as anterior interbody fusion (ALIF) and oblique interbody fusion (OLIF), have been proposed and widely used in lumbar degenerative diseases [2]. These approaches provide direct access to the vertebral column, thereby effectively avoiding injury to the posterior muscle [3]. Moreover, ALIF and OLIF are applicable to improve endplate preparation for arthrodesis, shorten operation time, and reduce amount of blood loss compared with the traditional posterior approaches [4, 5]. Although these two approaches cannot realize direct neural decompression, they can achieve indirect decompression by restoring disc height, foraminal height, and foraminal area, and their clinical effects have been broadly confirmed [5].

ALIF and OLIF have their strengths and weaknesses. ALIF provides a direct visualization of the anterior column, allowing for a complete discectomy and a better endplate preparation, while it is associated with a higher risk of complications, such as vascular injury and neurological injury. Compared with ALIF, OLIF was reported with lower incidence rates of vascular injury, abdominal complications, and reverse ejaculation. However, the operative field is limited in OLIF with lumbar discectomy, and implementation of direct decompression is a surgical challenge. The postoperative segmental stability after ALIF and OLIF is an immediate concern of orthopaedic surgeons, which determines the clinical outcome for bone fusion or pseudarthrosis. Bilateral pedicle screws (PS) and anterior plate (AP) are mainly used to provide stability to promote fusion, and a growing body of evidence proved that ALIF or OLIF combined with a stand-alone (SA) cage may contribute to a satisfactory clinical outcome. To date, no conclusion has been reached on which surgical approach and what type of fixation system should be selected in terms of postoperative stability [6, 7].

Previous studies have mainly concentrated on comparing the effects of different supplementary fixations in ALIF and OLIF surgeries, while few studies have directly compared the ALIF and OLIF combined with supplementary fixations of the same type in biomechanics. The present study aimed to investigate the biomechanical response of ALIF and OLIF associated with different supplementary fixations (SA cage, integrated SA (ISA) cage, AP, and bilateral PS) using the finite element method. The results may provide a reliable basis for better understanding of differences between ALIF and OLIF and the effects of various fixation systems from the view of biomechanics.

## Materials and methods

### Development of an intact L4/5 lumbar spine model

Computed tomography (CT) images of the human lumbar spine were obtained from a 32-year-old man with no lumbar spinal disease. CT images were captured with 0.65-mm interval and saved as Digital Imaging and Communications in Medicine (DICOM) format. A three-dimensional finite element (3D-FE) model of L4–L5 lumbar spine was developed according to the collected CT images using Mimics 19.0 and 3-Matic 11.0 (Materialise, Inc., Leuven, Belgium) and Hypermesh 14.0 (Altair Technologies, Inc., Carlsbad, CA, USA) software. Briefly, CT images were imported to the Mimics 19.0 software to develop the contour of the skeletal component and intervertebral disc based on the grey threshold of each material. The skeletal component was then divided into three parts: cortical bone, cancellous bone, and posterior structure. The thickness of cortical bone was set to 0.5 mm. The developed components were then transported to 3-Matic 11.0 software and modified to make their contour close to the anatomy of the natural human lumbar spine. The intervertebral disc was divided into two parts: annulus ground and nucleus pulposus, according to the anatomical structure of the intervertebral disc. The facet joints with smooth articular surfaces and 0.5-mm gap were created using the offset and Boolean operation.

**Fig. 1:**
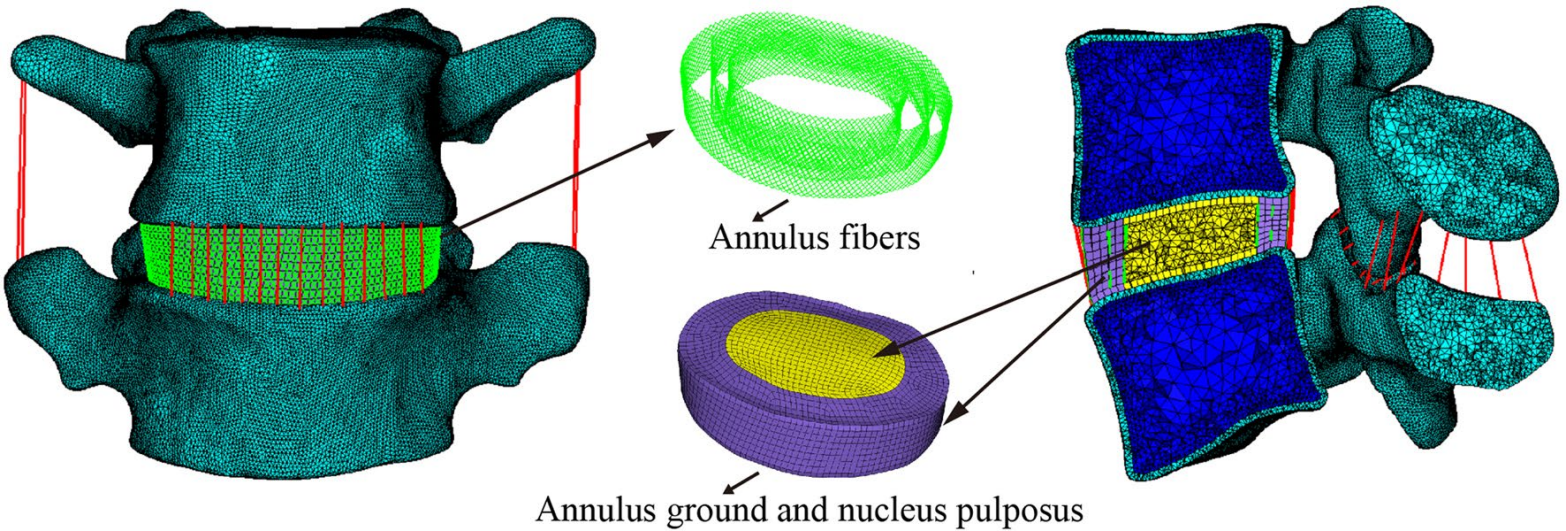
Finite element model of L4–L5 with associated soft tissues

The solid geometry models were then imported into Hypermesh 14.0 software for pre-treatment. The cortical, cancellous, and posterior structures were meshed with C3D4 element. The annulus ground and nucleus pulposus were meshed with C3D8RH element. Net structure composed of one-dimensional (1D) truss element (T3D2) with an inclination between 15° and 45° to the horizontal planes was created within the annulus ground to simulate the annulus fibres [8]. The cartilaginous endplates and articular surfaces were constructed using 0.4-mm-thick shell element (S3). Seven major ligaments were simulated using T3D2 element with no compression stress, including anterior and posterior longitudinal ligaments, flavum ligaments, intertransverse ligaments, supraspinal ligaments, interspinous ligaments, and capsular ligaments. The interfaces among cortical bone, cancellous bone, posterior structure, nucleus pulposus, and annulus ground were set as tie constraints. The interactions between the articular surface of the facet joint and surface contact were set as node with a friction coefficient of zero. Thus, an intact L4/5 lumbar spine FE model was successfully created (Fig. 1). Each component in this model was assigned corresponding material parameters, as shown in Table 1.

**Table 1:** Material properties of the intact lumbar spine model and ALIF/OLIF surgical models

Components	Young’s modulus (MPa)	Poisson’s ratio	Cross-sectional area (mm^2^)
Cortical	12,000	0.3	NA
Cancellous	100	0.3	NA
Endplate	1000	0.3	NA
Posterior structure	3500	0.3	NA
Bone graft	3500	0.3	NA
Facet cartilage	10	0.4	NA
Annulus fibre	450	0.45	NA
Implants	110,000	0.3	NA
Nucleus pulpous	Mooney–Rivlin, C10 = 0.12, C01 = 0.03		NA
Annulus ground	Mooney–Rivlin, C10 = 0.18, C01 = 0.045		NA
Anterior longitudinal	7.8 (< 12), 20 (> 12%)		63.7
Posterior longitudinal	10 (< 11%), 20 (> 11%)		20
Ligamentum flavum	15 (< 6.2%), 19.5 (> 6.2%)		40
Capsular	7.5 (< 25%), 32.9 (> 25%)		30
Interspinous	10 (< 14%), 11.6 (> 14%)		40
Supraspinous	8.0 (< 20%), 15 (> 20%)		30
Intertransverse	10 (< 18%), 58.7 (> 18%)		1.8

### Development of ALIF and OLIF models

**Fig. 2:**
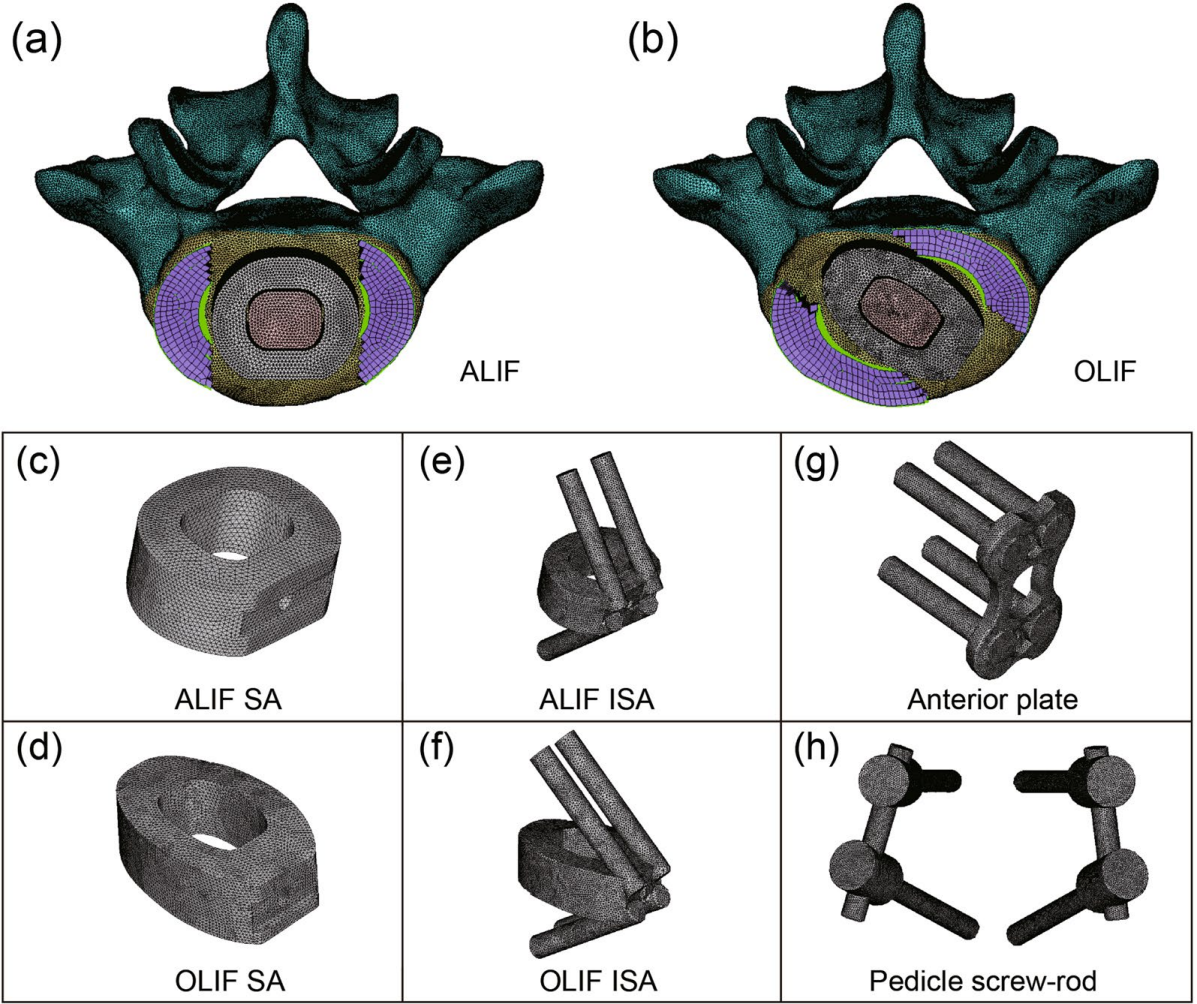
Schematic diagram of cage implantation position in the ALIF and OLIF surgical models and all the designed fixation systems

**Fig. 3:**
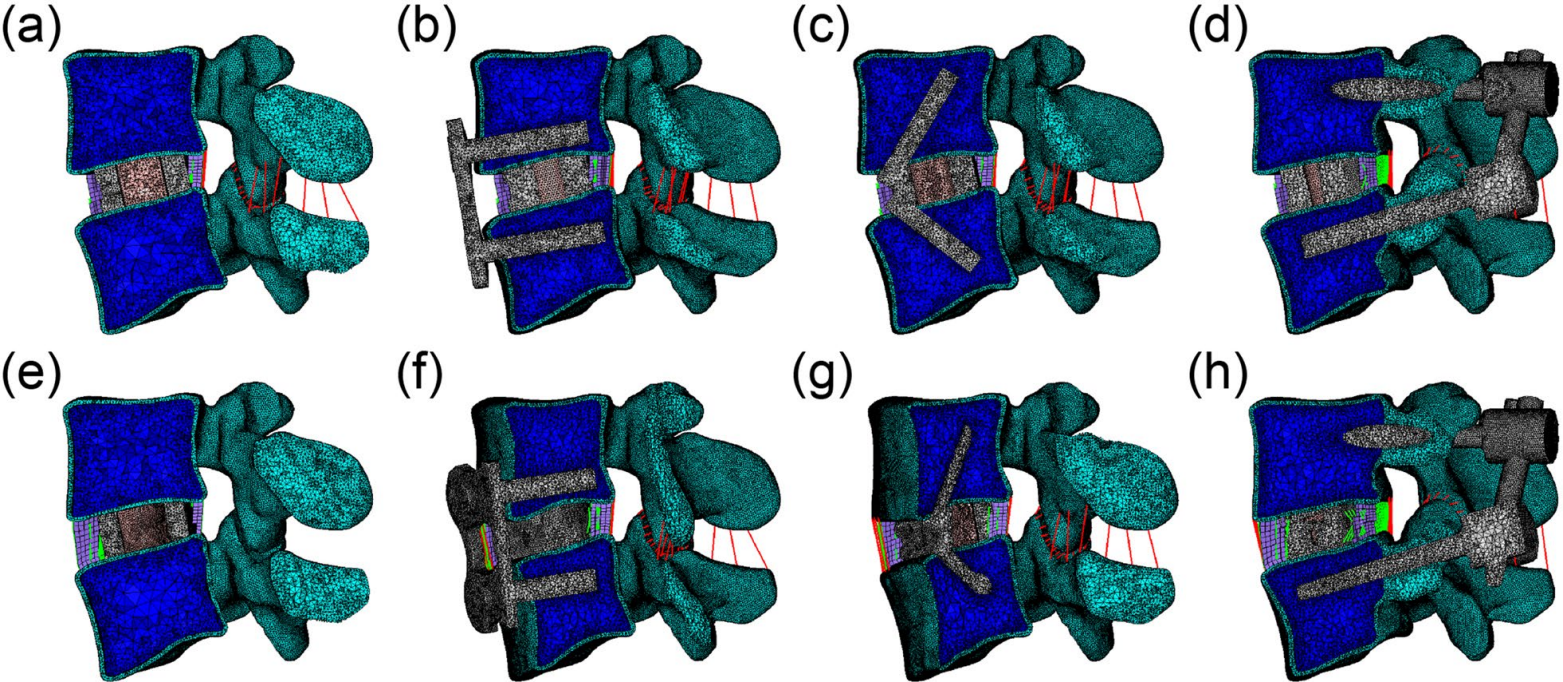
Schematic diagram of the ALIF and OLIF surgical models. **a** ALIF combined with stand-alone cage, **b** ALIF combined with anterior plate, **c** ALIF combined with integrated stand-alone cage, **d** ALIF combined with pedicle screw, **e** OLIF combined with stand-alone cage, **f** OLIF combined with anterior plate, **g** OLIF combined with integrated stand-alone cage, **h** OLIF combined with pedicle screw

In the present study, SA cage, ISA cage, AP system, and PS system were designed using 3-Matic software, as shown in Fig. 2. The size of the SA cage used in ALIF and that of the SA cage used in OLIF was 25 × 20 mm^2^ and 28 × 18 mm^2^ in footprint, respectively. The height and the lordosis angle were designed to fit the endplate. The ISA cage was designed by adding four integrated screws. The AP system was composed of a 40-mm length plate and four screws with diameter of 5.5 mm. The PS system comprised two rods and four PSs with diameter of 6.5 mm. The intervertebral disc of the intact L4/5 model was partially removed according to the OLIF and ALIF procedures, as shown in Fig. 2a, b. The SA cage, ISA cage, AP system, or PS system was then inserted separately. A bone graft with an appropriate size was inserted into the cage. The key point is the angle between the cage and the coronal plane. The cages were placed in parallel to the coronal plane in the ALIF model and with the angle of 15° in OLIF model according to postoperative CT images. Finally, eight ALIF and OLIF models were developed in the present study, including ALIF/OLIF SA (SA cage was implanted), ALIF/OLIF ISA (ISA cage was implanted), ALIF/OLIF AP (SA cage and AP were implanted), and ALIF/OLIF PS (SA cage and bilateral PS were implanted), as depicted in Fig. 3.

The implants were meshed with C3D4 element and were assigned as titanium material. According to actual surgery, the anterior longitudinal ligament was removed in all ALIF models, while retained in all OLIF models. The interfaces among endplate, cage, and bone graft were defined as nonlinear contact with a friction coefficient of 0.07. The vertebra-screw interfaces and screw-implant interfaces were assigned as tie constraint. The other pre-treatments of ALIF and OLIF models were the same as those of the intact L4/5 model.

### Boundary and loading conditions

The developed models were imported into Abaqus 6.13 software (Simulia, Inc., Johnston, RI, USA) for analysis. For the intact L4/5 model, the inferior endplate of L5 was fixed at 6 degrees of freedom, and 10 Nm of pure moment was applied to the superior endplate of L4 to simulate flexion, extension, lateral bending, and rotation. The L4/5 segmental ranges of motion (ROMs) were compared with data of a cadaver study carried out by Panjabi et al*.* [10] and a FE study conducted by Matsumoto et al*.* [9]. For each of the ALIF and OLIF models, the inferior endplate of L5 was totally constrained. A follower compressive load of 400 N was applied to the superior endplate of L4, and 2 and 10 Nm of pure moment were then applied, respectively.

## Results

### Model validation

**Fig. 4:**
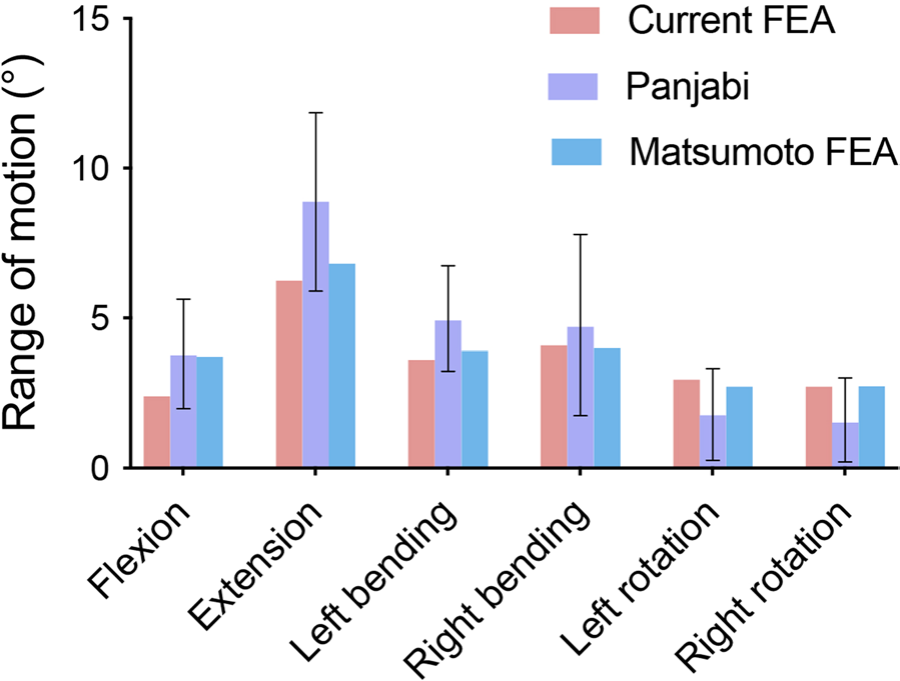
Comparison of the ROM data in the intact L4–L5 spinal model and experimental data from Panjabi et al. [10] and finite element data from Matsumoto et al. [9].2

The ROM of L4–L5 in flexion, extension, left lateral bending, right lateral bending, left rotation, and right rotation was 2.40°, 6.24°, 3.61°, 4.09°, 2.95°, and 2.72°, respectively. In all the postures, the ROM of L4–L5 fell within the range of in vitro experimental values reported by Panjabi et al*.* [10] and was very close to the results of the FE study carried out by Matsumotoet et al*.*[9]. The validation results of the intact L4/5 model are shown in Fig. 4. The developed intact L4/5 model could be used in the modelling and FE analysis of ALIF and OLIF.

### ROM of the surgical segment

**Fig. 5:**
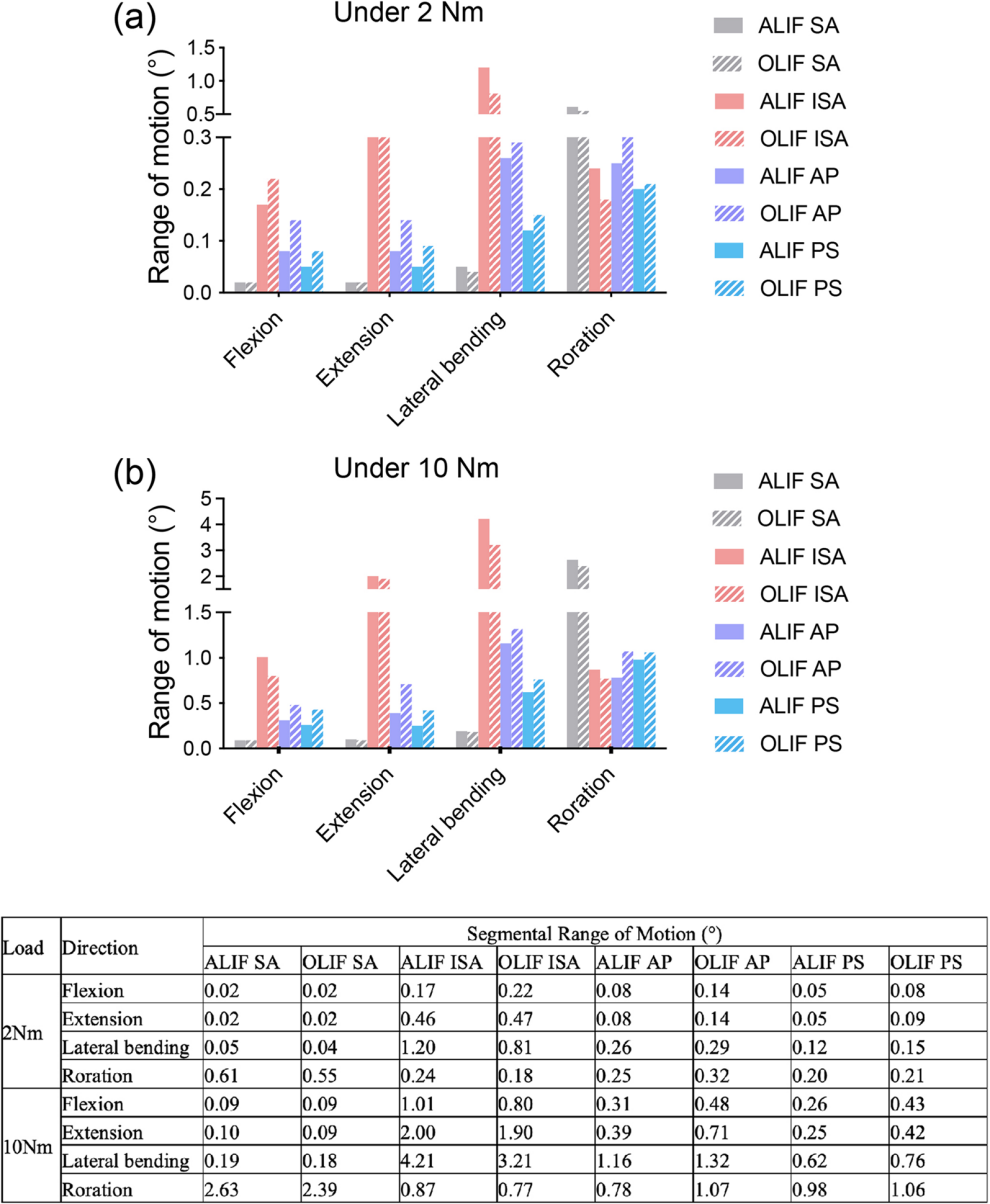
Segmental ROM of ALIF/OLIF models subjected to hybrid loads. ALIF SA: ALIF combined with stand-alone cage. ALIF ISA: ALIF combined with integrated stand-alone cage. ALIF AP: ALIF combined with anterior plate. ALIF PS: ALIF combined with pedicle screw. OLIF SA: OLIF combined with stand-alone cage. OLIF ISA: OLIF combined with integrated stand-alone cage. OLIF AP: OLIF combined with anterior plate. OLIF PS: OLIF combined with pedicle screw. The table listed the values of segmental ROM of ALIF/OLIF models subjected to hybrid loads

Eight surgical models, including ALIF/OLIF SA, ALIF/OLIF ISA, ALIF/OLIF AP, and ALIF/OLIF PS were eventually developed successfully, as shown in Fig. 3. The values of the segmental ROM are shown in Fig. 5. The ROM of the L4–L5 segment decreased in all the surgical models compared with that in the intact L4/5 model. When different surgical methods and additional fixation systems were used, the ROM significantly changed. Under 2 Nm moment, ALIF/OLIF SA showed the lowest ROM in flexion, extension, and lateral bending, while the highest ROM in axial rotation. Conversely, ALIF/OLIF ISA presented the highest ROM in flexion, extension, and lateral bending. In each posture, AP induced a higher ROM than posterior fixation no matter ALIF or OLIF was used. Meanwhile, the OLIF always showed a higher ROM than ALIF in each posture when the AP or PS was used. When 10 Nm moment was applied, the ROM of each model was larger than that induced by 2 Nm moment. The ROM showed a similar variation trend among models as that induced by 2 Nm moment, in which except for that in axial rotation, AP induced a lower ROM in ALIF than that in OLIF, and PS presented a similar ROM in both ALIF and OLIF.

### Maximum stress on endplate

**Fig. 6:**
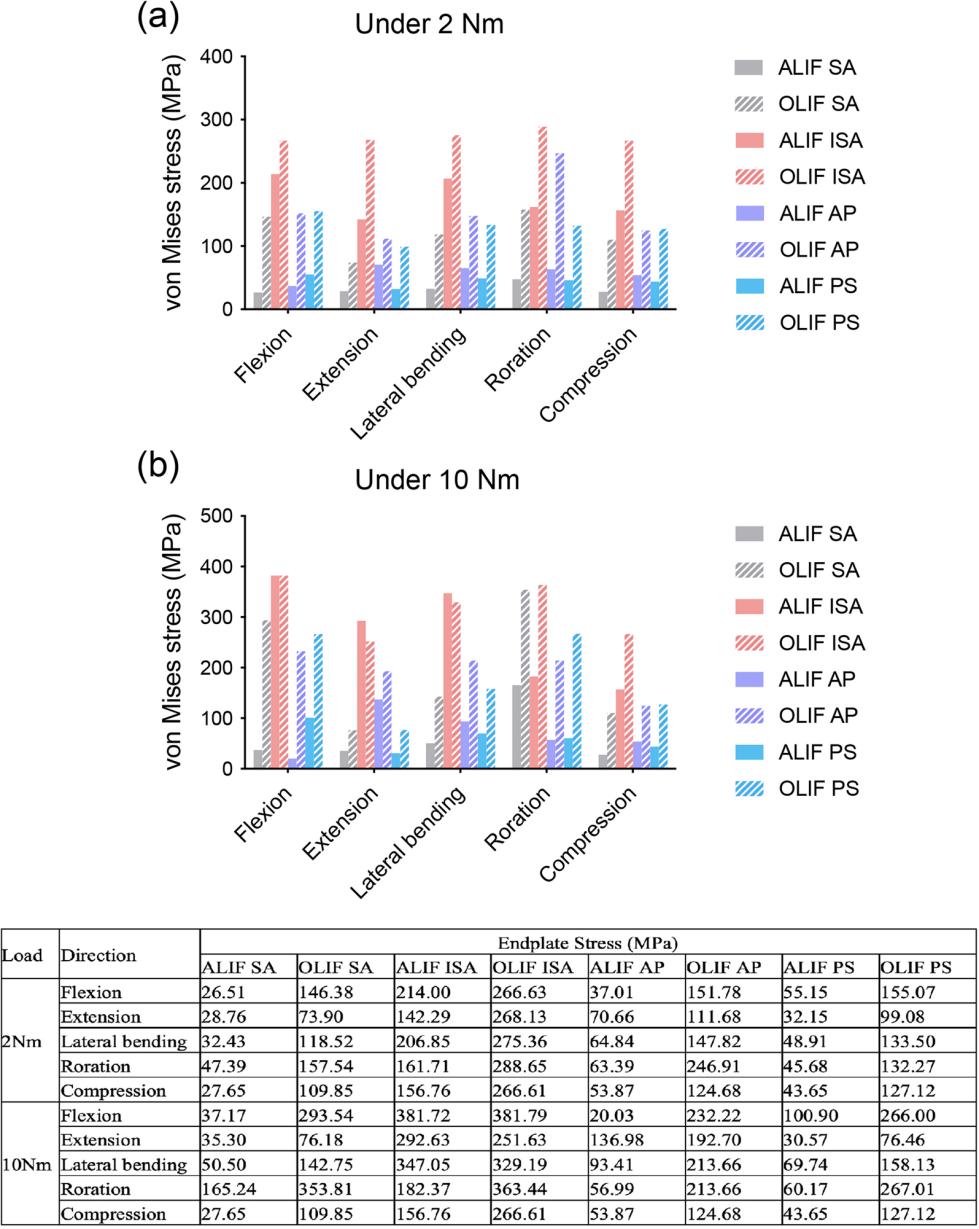
Endplate stress of ALIF/OLIF models subjected to hybrid loads. ALIF SA: ALIF combined with stand-alone cage. ALIF ISA: ALIF combined with integrated stand-alone cage. ALIF AP: ALIF combined with anterior plate. ALIF PS: ALIF combined with pedicle screw. OLIF SA: OLIF combined with stand-alone cage. OLIF ISA: OLIF combined with integrated stand-alone cage. OLIF AP: OLIF combined with anterior plate. OLIF PS: OLIF combined with pedicle screw. The table listed the values of endplate stress of ALIF/OLIF models subjected to hybrid loads

The stress distribution on the endplates significantly changed when different surgical methods and fixation systems were used, as shown in Fig. 6. The ALIF models showed a lower stress than the OLIF models in almost all postures, except for in extension and lateral bending under 10 Nm moment. When 2 Nm moment was applied, the ALIF SA model showed the lowest stress, and the ALIF ISA model exhibited the highest stress in all postures among ALIF models. The ALIF PS model presented a lower stress than ALIF AP in the majority of postures, except for in flexion. The OLIF ISA model also showed the highest stress in all postures among OLIF models. The OLIF SA model had the lowest stress in flexion, extension, lateral bending, and under compression, while showed a higher stress than OLIF PS in rotation. The stress of OLIF PS was lower than that of OLIF AP in extension, lateral bending, and rotation. When 10 Nm moment was applied, the ALIF ISA and OLIF ISA models still had the highest stress in all postures. The ALIF SA showed the lowest stress in lateral bending and under compression. In flexion and rotation, the lowest stress was found in the ALIF AP model, while in extension, the ALIF PS model exhibited the lowest stress. The ALIF AP model presented a higher stress than ALIF PS in extension, lateral bending, and under compression, which was opposite in flexion and rotation. The OLIF SA model showed the lowest stress in extension, lateral bending, and under compression, and the OLIF AP model exhibited the lowest stress in flexion and rotation. The values of the endplate stress are listed in Fig. 6.

### Stress on screw-vertebra interface

**Fig. 7:**
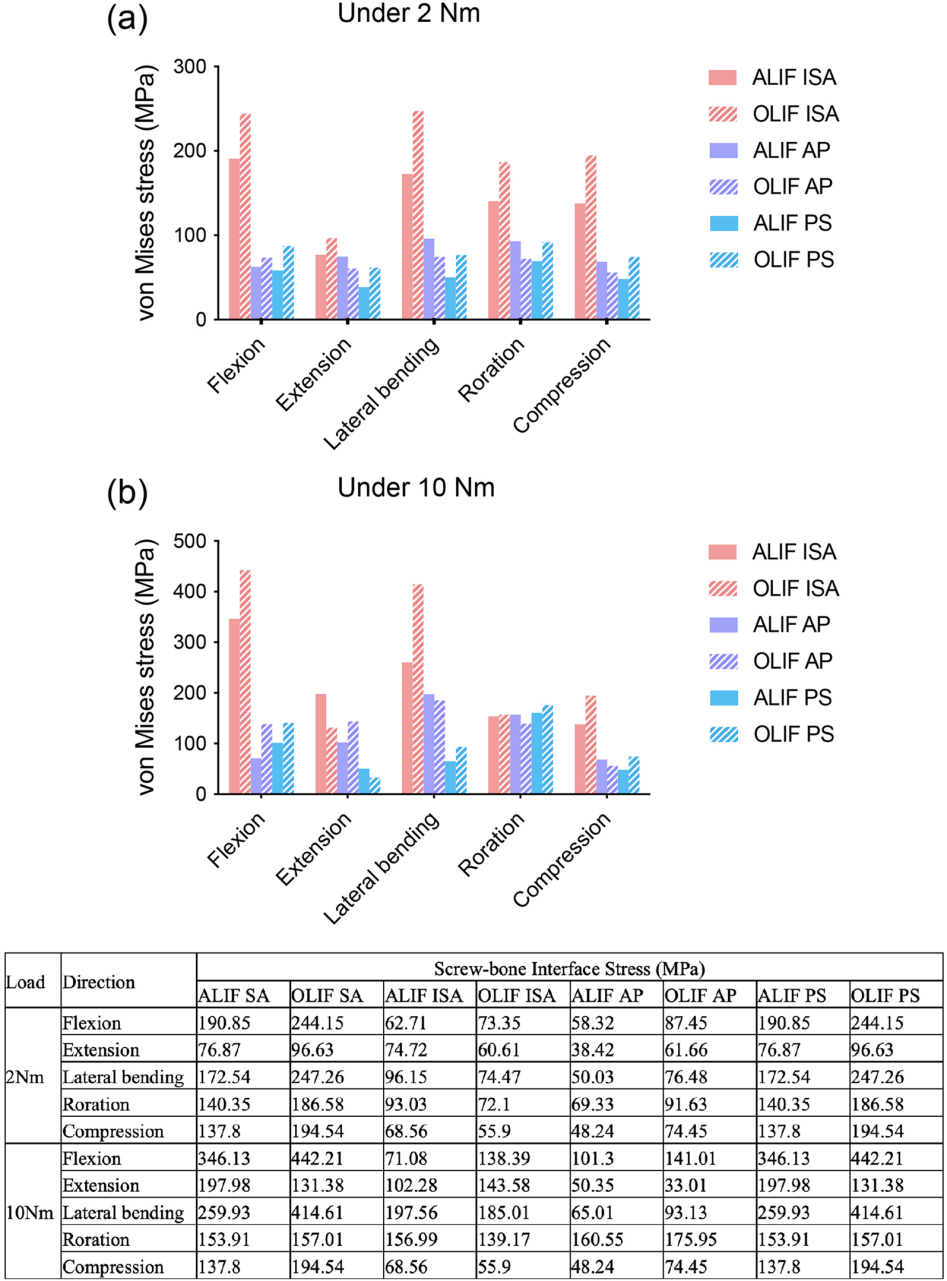
Screw-bone interface stress of ALIF/OLIF models subjected to hybrid loads. ALIF SA: ALIF combined with stand-alone cage. ALIF ISA: ALIF combined with integrated stand-alone cage. ALIF AP: ALIF combined with anterior plate. ALIF PS: ALIF combined with pedicle screw. OLIF SA: OLIF combined with stand-alone cage. OLIF ISA: OLIF combined with integrated stand-alone cage. OLIF AP: OLIF combined with anterior plate. OLIF PS: OLIF combined with pedicle screw. The table listed the values of screw-bone interface stress of ALIF/OLIF models subjected to hybrid loads

For different models and loads, the maximum stress on the screw-vertebra interface is shown in Fig. 7. No screw was used in ALIF/OLIF SA models; thus, no data were correspondingly listed. While 2 Nm moment was applied, the ALIF models showed less screw-bone interface stress than OLIF models in the majority of planes, except for when AP was used. The ALIF SA model presented the largest screw-vertebra interface stress in each loading plane, followed by ALIF AP and ALIF PS models. For OLIF models, the screw-vertebra interface stress of the OLIF ISA model also was the highest in all postures, and that of the OLIF AP model was the lowest in all planes. When 10 Nm moment was applied, the ALIF and OLIF models showed less screw-vertebra interface stress in different planes when different fixation systems were used, and no significant differences were found. The ALIF ISA model presented the highest screw-vertebra interface stress in flexion, extension, and lateral bending. In rotation, the ALIF ISA, ALIF AP, and ALIF PS models showed a similar screw-vertebra interface stress. The OLIF ISA model exhibited the largest screw-vertebra interface stress in flexion and lateral bending. The screw-vertebra interface stress of ALIF/OLIF AP models was lower than that of ALIF/OLIF PS in flexion and rotation, while higher in extension and lateral bending. The values of the screw-bone interface stress are listed in Fig. 7.

### Cage migration in ALIF/OLIF SA models

**Fig. 8:**
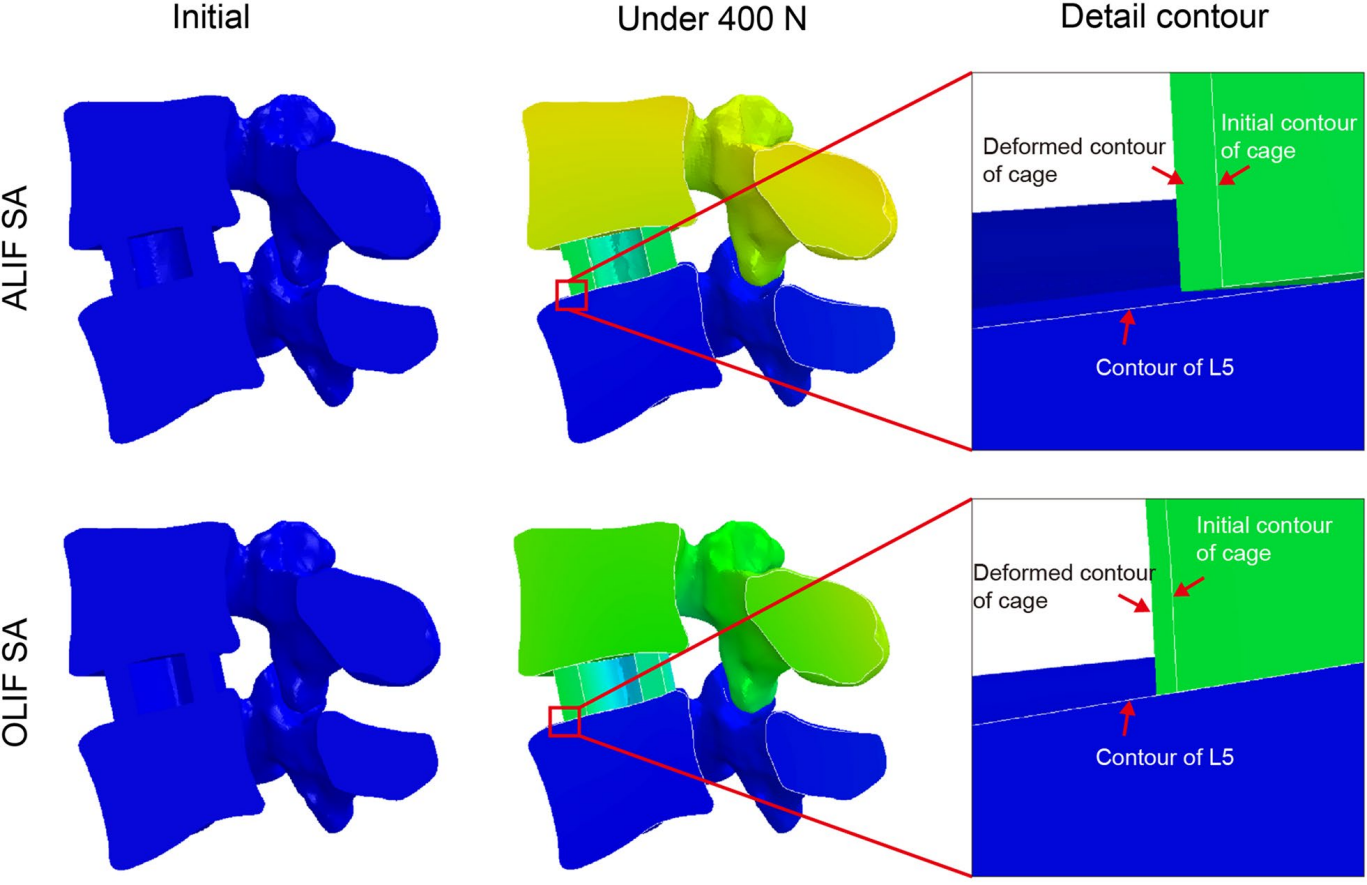
Schematic representation of the cage migration of the ALIF SA and OLIF SA models. ALIF SA: ALIF combined with stand-alone cage. OLIF SA: OLIF combined with stand-alone cage

Figure 8 shows the positions of L4/L5 and the cage in ALIF SA and OLIF SA models under initial condition and a follower load of 400 N. The red arrows indicated the contour of the superior L5 endplate and the initial and deformed contours of the interbody cage. Apparent anterior slip of cage relative to L5 superior endplate was found in these two models. No similar cage displacement was found in other models.

## Discussion

The present study assessed the biomechanical stability, the risk of cage subsidence, and screw loosening incidence of ALIF and OLIF accompanied with various supplementary fixation systems, including ALIF/OLIF SA, ALIF/OLIF ISA, ALIF/OLIF AP, and ALIF/OLIF PS. The results suggested that when the AP and the PS were used, ALIF provided greater segmental stability and less endplate stress, as well as less screw-vertebra interface stress under lower loading compared with OLIF. Using a SA cage without integrated screw fixation in ALIF and OLIF was associated with cage migration. Addition of integrated screws into a SA cage limited the migration of the cage, while did not provide preferable segmental stability in flexion, extension, and lateral bending. Furthermore, the ISA cage induced remarkably endplate stress and screw-bone interface stress. Besides, AP and PS provided higher stability and less endplate/screw-bone interface stress than integrated interbody cage in the majority of planes.

As the anterior lumbar interbody fusion was first introduced by Carpener in 1932 [11], it has been modified, optimized, and incorporated into various surgical methods. Among them, ALIF and OLIF are the most widely used methods. Although they fall into the anterior approach, ALIF and OLIF differ in terms of advantages and disadvantages. Numerous studies have compared ALIF and OLIF in terms of clinical results; however, few biomechanical perspective studies were conducted. The present study showed that when the PS and AP were used, OLIF models presented higher ROM and endplate/screw-bone interface stress than ALIF in the most of directions. These results indicated that ALIF is more beneficial to postoperative segmental stability and less susceptible to cage subsidence and internal fixation loosening. David et al*.* systematically reviewed the literature on ALIF and OLIF, and they found that radiographic subsidence occurred in 30% in OLIF and only 10.2% in ALIF [2], which can be well-explained by the higher endplate stress in OLIF models found in the current study. This difference in endplate stress was attributed to the smaller length and surface area of OLIF than that of ALIF [2]. However, the cages used in ALIF and OLIF in the present study shared similar surface areas in footprint. The discrepancies might be related to different positions of the cage placed on the endplate. To simulate the limitations of ALIF and OLIF surgeries, the cage was placed in parallel to the coronal plane in ALIF models, whereas possessed a 15-degree angle in OLIF models. This position might result in the reduction in contact surfaces between the cage and endplate under motion. This point of view has been proposed by a previous study on PLIF [12], whereas no experiment has assessed the influences of position and shape of the cage on the biomechanical and clinical results. When the ISA cage was used, the ALIF models showed less endplate/screw-bone interface stress, while presented higher segmental ROM than OLIF models. This indicated that the ISA cage could not provide enough stability as the oblique-placed integrated cage. These results suggested placing the interbody cage in parallel to the coronal plane in surgery. Future research is needed to obtain more details on the effects of the interbody cage position on the stability and stress distribution.

Using a SA cage to achieve interbody fusion is very attractive due to its safety and simplicity. Numerous studies have recently reported the feasibility and satisfactory clinical outcomes of this technique in ALIF and OLIF [13–16]. The lowest ROM was found in ALIF/OLIF SA models in flexion, extension, and lateral bending in the present study. However, the ROM of ALIF/OLIF SA models in rotation was significantly higher than that of other models. This result was consistent with that reported by Martin in an in vitro biomechanical experiment [6]. In addition, a noticeable cage migration was found in ALIF/OLIF SA models when the follower load of 400 N was applied (Fig. 8). Given that the follower load of 400 N was applied to simulate the normal 400 N follower load weight, the loading process could be regarded as the transition from a lying position to a standing position. This indicates that once patients stand up or lie down after surgery, they may suffer from a cage migration. This situation is obviously detrimental to the immediate postoperative stability and interbody fusion. Cage migration has also been reported in several previous studies [6, 17–19]. Martin et al*.* reported a 25% incidence of cage migration in ALIF SA surgery in the biomechanical analysis [6]. Wendy et al. reported some clinical cases of cage migration in OLIF SA surgery [18, 19]. The micromotion in the bone-cage interface was regarded as one of the primary reasons for the pseudarthrosis and cage subsidence [20, 21]. As frequently reported previously, the ALIF/OLIF SA induced a significantly lower fusion rate, while a higher incidence of complications than ALIF/OLIF combined with AP or PS system [22, 23]. Thus, using a SA cage in ALIF or OLIF surgery may not be a promising approach for interbody fusion.

The present study found no cage migration in ALIF ISA and OLIF ISA models. This finding explained why ALIF/OLIF combined with an ISA cage always showed a greater stability and a higher fusion rate than the ALIF/OLIF SA surgery [6]. Adding additional integrated screws into the SA cage has been developed as an alternative method to improve the postoperative stability and retain the operative simplicity of the ALIF/OLIF SA surgery [6, 17, 24, 25]. However, it is essential to indicate whether the ISA cage can provide sufficient stability comparable to the PS system. The answer appears to be negative in the current study. The results revealed that the ALIF/OLIF ISA models showed significantly higher ROMs in flexion, extension, and lateral bending than ALIF/OLIF AP and ALIF/OLIF PS. In addition, ALIF/OLIF ISA models induced significantly higher endplate stress and screw-bone interface stress than ALIF/OLIF AP and ALIF/OLIF PS. The results also indicated that the ISA cage improved the stability, while did not improve as strong enough as AP and PS. The high rates of cage subsidence and screw loosening are predictable because of the increased stress. A systematic review carried out by Manzur et al*.* [26] found that high-grade subsidence was the most common complication of an ISA cage, with a rate of 11.1%. Lei et al*.* compared imaging and clinical results of ALIF with those of ISA cage and TLIF and found that the former technique exhibited a significantly higher rate of cage subsidence [27]. The rate of cage subsidence varied in different studies using an ISA cage without additional fixation instruments, and the reported fusion rates are acceptable. This might be related to the fact that patients undergoing ALIF/OLIF with an ISA cage were mainly screened carefully. For instance, patients with a low bone mineral density were not involved in previous clinical studies. Considering the relatively lower stability and the higher risk of cage subsidence and screw loosening, the future research should concentrate on improving the architectural design to optimize the stress distribution and develop detailed indications for ALIF/OLIF with an ISA cage.

In general, the PS system was considered as the gold standard, providing segmental stability in lumbar surgery. However, it has several disadvantages, including longer duration of fluoroscopy, increased blood loss, longer operation time, and paraspinal muscle injury. The AP is expected to supply identical segmental stability simultaneously to avoid drawbacks. Numerous studies combined ALIF/OLIF with the AP to treat lumbar degenerative diseases and reported satisfactory clinical results; however, few experiments have investigated its biomechanical characteristics. The present study found that except for rotation, the postoperative segmental ROM induced by the AP was larger than that caused by the PS in ALIF and OLIF. This result was highly consistent with that reported by previous in vitro cadaveric biomechanical studies [6, 28, 29]. The endplate stress and the screw-bone stress induced by the AP were higher than those by the PS; however, the difference was relatively small, and it showed the opposite result to some extent. These results suggested that the AP could not provide identical stability as the PS. At present, the disparity is not remarkable to lead to significant differences in clinical effects, such as the rate of cage subsidence and internal fixation loosening. A great number of studies used ALIF/OLIF combined with the AP to treat various lumbar degenerative diseases, and reported the identical clinical result to that of the PS; no significant difference was found in the incidence of complications in clinic [30–32]. Furthermore, the AP combined with ALIF/OLIF has been proved to surpass the PS in terms of the amount of blood loss, operation time, soft tissue disruption, and minimizing the exposure [33]. Accordingly, it can be inferred that ALIF/OLIF combined with the AP can be used safely and efficiently in lumbar interbody fusion surgery. Further attention should be paid to develop appropriate indications to avoid the possible vertebral fracture and cage subsidence.

There were some limitations in the present study. First, the models were developed based on medical images of a healthy volunteer without any lumbar spine pathology. Second, to reduce excessive calculation, only one spinal unit was considered. Third, as part of a project in developing newly designed 3D-printed titanium interbody cage, the titanium property rather than polyether ether ketone (PEEK) was assigned to the cages employed in the current study. Although the pre-experiment suggested that this did not affect the above-mentioned results, readers should carefully use the results in PEEK cage. Fourth, the models were simplified to some extent. For instance, the bone graft and cage were designed as a uniform structure and specific details were neglected, such as the porosity of the graft bone and the sawtooth shape on the cage. The screws were set as solid cylinders and bound to the plate, rod, or cage; the interactions between screws and other components were set as tie constraint. These simplifications reduced the calculation cost and improved the convergence; however, they unavoidably affected the calculation accuracy. At present, the objective of a finite element study is to provide a general trend. The present study successfully calculated the differences in stability and stress distribution of ALIF/OLIF supplemented with various fixation systems under experimental conditions.

## Conclusions

The present study aimed to compare ALIF and OLIF surgeries and determine the effects of various supplementary fixation systems from the view of biomechanics. The results indicated that the AP and PS were associated with greater stability and less risk of subsidence and fixation loosening than other fixation systems. When the AP and PS were used, ALIF performed better in terms of postoperative stability and stress distribution in the endplate and screw-bone interface than OLIF. Using a SA cage in ALIF or OLIF surgery without any other fixation systems may lead to cage migration. Adding integrated screws into a SA cage in ALIF and OLIF surgery prevented cage migration, while it led to the insufficient stability and the risk of subsidence and fixation loosening. These findings could provide new insights into upgrading fixation systems in ALIF and OLIF surgeries and promote the safe application of these surgical methods.

## Data Availability

The datasets used and/or analysed during the current study are available from the corresponding author on reasonable request.

## Abbreviations

ALIF: Anterior lumbar interbody fusion
OLIF: Oblique lumbar interbody fusion
ROM: Range of motion
EPS: Endplate stress
SBIS: Screw-bone interface stress
PLIF: Posterior lumbar interbody fusion
TLIF: Transforaminal lumbar interbody fusion
CT: Computed tomography
FE: Finite element
SA: Stand-alone
ISA: Integrated stand-alone
AP: Anterior screw-plate
PS: Posterior pedicle screw-rod
